# b-AP15 enhances TRAIL-induced cell death in HNSCC via the induction of ROS/JNK/DR5 signalling

**DOI:** 10.1038/s41417-026-01038-3

**Published:** 2026-05-02

**Authors:** Fin T. A. Brown, Lucy Quesne, Louisa M. Wootton, Holly Foxell, Ipek Erseven, Emilia Ewen Benns, Molly Tate, Ethan L. Morgan

**Affiliations:** 1 School of Life Sciences, University of Sussex, Brighton, UK; 2 Present Address: Tumour Virology Group, The Cyprus Institute of Neurology and Genetics, Nicosia, Cyprus

**Keywords:** Head and neck cancer, Cell biology

## Abstract

Therapeutic resistance to chemotherapy or radiotherapy is a significant issue in several cancers, including head and neck squamous cell carcinoma (HNSCC). One pathway associated with therapeutic resistance is the NFκB pathway, which promotes survival in response to the cytokine TNFα, a key mediator of chemotherapy and radiotherapy-induced cytotoxicity. However, direct targeting of the NFκB pathway is associated with significant toxicity and thus targeting the regulation of this pathway is a promising therapeutic target. We recently demonstrated that the USP14/UCHL5 inhibitor b-AP15 inhibits NFκB activity, inhibiting proliferation and inducing apoptosis in HNSCC cells. Furthermore, b-AP15 treatment sensitised HNSCC cells to the cytotoxic effects of TNFα, as well as TNF-inducing radiation treatment. Here, we investigated if b-AP15 sensitised HNSCC cells to tumor necrosis factor-related apoptosis-inducing ligand (TRAIL), a cancer selective member of the TNF family. b-AP15 treatment sensitised HNSCC cells to TRAIL treatment. Mechanistically, we show that b-AP15 induced expression of the TRAIL receptor Death Receptor 5 (DR5)/TRAIL Receptor 2 (TRAILR2), which was required for b-AP15-mediated TRAIL sensitisation. b-AP15 induced reactive oxygen species (ROS) and activated the JNK signalling pathway and both ROS and JNK signalling were required for the induction of DR5 expression and TRAIL sensitisation. We further show that b-AP15-mediated reduction of the NFκB-dependent gene XIAP induced DR5 expression and TRAIL sensitisation and that combination between b-AP15 and IAP antagonists was synergistic in HNSCC cells in vitro. Our data further define the mechanism of b-AP15-mediated cytotoxicity and highlight potential combination treatments that warrant further exploration in pre-clinical studies in HNSCC.

## Introduction

Head and neck squamous cell carcinoma (HNSCC) is the seventh most common cancer worldwide, with over 900,000 new cases and 450,000 deaths worldwide per year [1]. HNSCCs include a range of cancers of the head and neck area, including cancers of the lip, oral cavity, larynx and hypopharynx [1]. HNSCC has been primarily associated with alcohol and tobacco use; however, an increase in human papillomavirus positive HNSCC (HPV + HNSCC) has been observed over the past two decades [2]. These two sub-types of HNSCC have significantly different prognoses, with the five-year survival rate ranging between 28.8% and 58.7% for HPV- HNSCC and 52.2% and 77.6% for HPV-positive HNSCC, depending on the tumor subsite. Currently, standard treatment options include surgery, chemotherapy and radiotherapy, with limited treatment options for recurrent, metastatic HNSCC [2]. Furthermore, the side effects of standard treatments are often debilitating, necessitating the need for new treatment options that improve the survival outcomes in HPV- HNSCC to standard therapies, and aimed at de-escalating treatment-associated toxicities in HPV + HNSCC [3, 4]. The cytotoxic effects of chemotherapy and radiotherapy are partially associated with the inflammatory cytokine TNFα [5, 6]. This cytokine induces cell death through multiple mechanisms but is often circumvented via activation of the NFκB pathway, which is a critical regulator of survival and therapeutic resistance in several cancers [7–9]. In HNSCC, the NFκB pathway promotes resistance to cytotoxic chemotherapy and radiotherapy [10–12]. It is aberrantly activated via genomic alterations (gain or activating mutations of *PIK3CA* in HPV- HNSCC, or loss of *TRAF3*/gain of *HRAS* and loss of *CASP8* in HPV + HNSCC) or by the HPV oncoprotein E6 [13–15]. However, targeting the NFκB pathway direct is highly toxic and thus alternative ways of targeting this pathway are needed. We previously demonstrated that the deubiquitinating enzyme USP14 promotes NFκB activity in HNSCC cells [16]. Inhibition of USP14 via the small molecule b-AP15 prevents TNF-induced nuclear translocation of the NFκB subunit RelA by preventing the proteasomal degradation of IκBα in a ubiquitin-dependent manner. Furthermore, b-AP15 treatment promoted TNF and radiation-induced cell death, both in vitro and in vivo [16]. Whilst we demonstrated the ability of b-AP15 to sensitise HNSCC cells to TNFα, whether it can also sensitise HNSCC cells to other so-called ‘death ligands’ is unclear.

Tumor necrosis factor (TNF)-related apoptosis-inducing ligand (TRAIL) is a member of the TNF family [17]. TRAIL is produced by several immune cells, including cytotoxic T cells and natural killer (NK) cells, and plays a key role in tumour immune surveillance. Importantly, TRAIL preferentially induces apoptosis in cancer cells, making it more selective as a potential therapeutic target than TNFα [17, 18]. Through binding to the TRAIL receptors TRAILR1/Death Receptor 4 (DR4) and TRAILR2/DR5, TRAIL induces apoptosis through Fas-associated death domain (FADD)-dependent recruitment and activation of caspase-8, termed the Death-inducing Signalling Complex (DISC). This can be inhibited by FLICE-inhibitory protein (c-FLIP), which competes with caspase-8 to bind to FADD at the DISC, or via Cellular Inhibitors of Apoptosis (cIAP) proteins, which prevent caspase activation [19]. Another IAP family member, X-linked IAP (XIAP), also contributes to inhibition of cell death via caspase inhibition [20].

Here, we demonstrate that b-AP15 sensitises HNSCC cells to TRAIL via the upregulation of the TRAIL receptor DR5. Mechanistically, this occurs via the induction of reactive oxygen species (ROS) and activation of the JNK/c-Jun pathway. We further show that reduction of the NFκB-dependent gene XIAP is required for b-AP15-mediated DR5 expression and TRAIL sensitisation. Finally, we show that combination treatment between b-AP15 and IAP antagonists are synergistic in HNSCC cells. Our findings further define the mechanism of b-AP15-mediated cytotoxicity in HNSCC cells and highlight potential combination treatments that warrant further exploration in pre-clinical studies.

## Methods and materials

### Cell lines

UMSCC1 cells were kindly provided by the Fanconi Anaemia Research Fund [21]. UMSCC22A cells were purchased from Merck (Germany). UMSCC47 cells were kindly provided by Prof Andrew Macdonald (University of Leeds, UK). Cells were negative for Mycoplasma during this investigation and cell identify was confirmed by STR profiling.

### Plasmids, siRNAs and reagents

pcDNA3-Xiap-Myc was a gift from Guy Salvesen and was purchased from Addgene (#11833) [22]. siRNA targetting DR5 were purchased from Qiagen (FlexiTube GeneSolution GS8795 for TNFRSF10B; SI03094063, SI03038665, SI00056707 and SI00056700).

The small molecule inhibitors b-AP15, JNK-IN-8, N-acetylcysteine (NAC), Xevinapant and Tolinapant were purchased from MedChemExpress (USA). 2′,7′-Dichlorodihydrofluororescein diacetate (H_2_DCFDA) was purchased from Abcam (UK). Recombinant human TRAIL (Gibco; USA) was purchased from Fisher Scientific (USA) and DR5/TRAILR2 agonist (MAB631, Bio-Techne; USA) was purchased from R&D Systems (USA). TRAIL, DR5/TRAILR2 agonist, inhibitor and siRNA concentrations used are listed in each figure legend.

### Transfections

Transfection of plasmid DNA was performed with a DNA to Lipofectamine® 2000 (ThermoFisher Scientific) ratio of 1:2.5. Transfection of siRNA was performed with a siRNA to Lipofectamine® 2000 ratio of 1:2. At the required time point, cells were harvested and processed as needed.

### Western blot

Cells were treated as indicated in the figure legends and lysates were generate as previously [23]. Equal amounts of protein from cell lysates were separated by SDS PAGE and transferred to a PVDF membrane. After blocking in Odyssey blocking buffer (LI-COR Biosciences; USA), membranes were incubated with primary antibodies overnight at 4°C. After washing, membranes were incubated with species-specific IRdye-conjugated secondary antibodies for 1 h at room temperature. The following antibodies were used at 1:1000 dilution unless otherwise stated: Phospho-SAPK/JNK (Thr183/Tyr185) (1:500 - 81E11; 4668, CST), SAPK/JNK (9252, CST), Phospho-c-Jun (Ser73) (1:500 - D47G9; 3270, CST), c-Jun (60A8; 9165, CST), SOD2 (1:500 – D3X8F; 13141, CST), XIAP (66800-1-Ig, Proteintech), cIAP1 (66626-1-Ig; Proteintech), Myc (9E10; sc-40, Santa Cruz Biotechnology (SCBT), USA) and GAPDH (G-9; sc365062, SCBT). Signal was visualised using LI-COR ODYSSEY Infrared Imaging System (LI-COR Biosciences).

### RNA extraction and RT-qPCR

Cells were treated as indicated in the figure legends. Cells were then trypsinised and centrifuged. The pellet was then resuspended in QIAzol Lysis reagent (Qiagen) and homogenised. RNA was then precipitated using isopropanol, washed in ethanol and redissolved in RNase-free water. RNA quality and concentration was assessed using a Nanodrop. RT-qPCR was performed using the one-step SyBr green RT-qPCR kit from Apto-Gen (UK) and analysed on a Quanto Studio 3 (Applied Bioscience; USA). The following primers were used, with *GAPDH* used as a control: *SOD2* (Forward (F) - 5’ ACGTCACCGAGGAGAGTACC 3’, Reverse (R) - 5’ TAGGGCTGAGGTTTGTCCAG 3’), *XIAP* (F - 5’ ATTCACTTGAGGAGTGTCTGGT 3’, R - 5’ ATGTCCTTGAAACTGAACCCCA 3’), *TNFRSF10A* (F - 5’ AGCCCTTTGGGAGTTGTG 3’, R - 5’ TGGTGCAGGGACTTCTCTCT 3’), *TNFRSF10B* F - 5’ GATTCAGAGCTCACAACGACC 3’, R - 5’ GGTGGGCTGCAAGATACTCA 3’) and *GAPDH* (F - 5’ GACAGTCAGCCGCATCTTCT 3’, R - 5’ GCGCCCAATACGACCAAATC 3’). Data was presented as relative to the control condition, and each condition was performed in triplicate.

### CCK8 viability assay

Cells were treated as indicated in the figure legends. After 48 h, CCK8 reagent (Proteintech; UK) was added per the manufacturer’s instructions and plates were read at 450 nm on a plate reader. Data was presented as relative to the control condition, and each condition was performed in triplicate.

### Colony formation assays

Cells were treated as indicated in the figure legends. At the indicated time point, cells were trypsinized and reseeded at low density and left to form colonies for 10-14 days as previous [24, 25]. Wells were then stained (1% crystal violet, 25% methanol) and visible colonies (≥50 cells) were counted manually. Data was presented as relative to the control condition, and each condition was performed in triplicate.

### Flow cytometry assays

For DR4 and DR5 analysis, cells were treated as indicated in the figure legends. At each time point, cells were trypsinised, collected by centrifugation and blocked in 5% BSA in PBS. Cells we then incubated with a PE conjugated DR4 (clone DJR1) or DR5 (DRJ2-4) antibody (1:100; BioLegend, USA) prior to analysis on a BD Cytoflex (BD Biosciences; USA). Gates were established using an isotype control antibody. Data from 10,000 cells per sample were analysed using Flow-Jo analysis software (Tree Star). Data was presented as relative to the control condition, and each condition was performed in triplicate. For detection of ROS, cells were treated as indicated in the figure legends. At each time point, cells were trypsinised, collected by centrifugation and resuspended in buffer containing the oxidation-sensitive dye, H_2_DCFDA (Abcam) prior to analysis on a BD Cytoflex (BD Biosciences). ROS levels were determined as the mean fluorescence intensity (MFI). Gates were established using a no dye negative control. Data from 10,000 cells per sample were analysed using Flow-Jo analysis software (Tree Star). Data was presented as relative to the control condition, and each condition was performed in triplicate. For Annexin V assay, cells were treated as indicated in the figure legends. At each time point, cells were then harvested by trypsinization and collected by centrifugation. Cells were resuspended and analysed using the CoraLite™488-Annexin V and PI Apoptosis Detection Kit (Proteintech) following the manufacturer’s instructions. Data from 10,000 cells per sample were analysed using Flow-Jo analysis software (Tree Star). Early apoptotic cells were defined as Annexin positive/PI negative and late apoptotic cells were defined as Annexin positive/PI positive [26]. Each condition was performed in triplicate.

### Statistical analysis

Combination index for cell viability assays was analysed for synergy using CompuSyn [27]. Drug synergy analysis, dose-response matrix experiments were analysed using the Bliss independence model via the SynergyFinder portal [28]. Data is presented as mean ± the standard deviation from a minimum of three biological replicates. Statistical significance was determined as follows: NS = not significant, *p < 0.05, **p < 0.01, ***p < 0.001 using an unpaired two-tailed Student’s t-test.

## Results

### b-AP15 enhances TRAIL-induced cell death in HNSCC cells

**Fig. 1:**
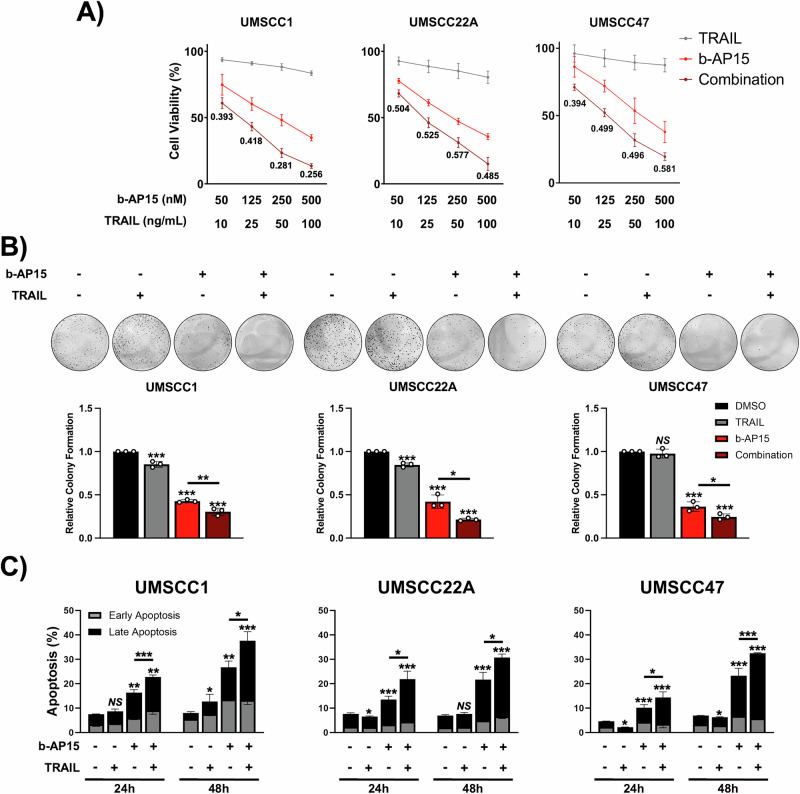
b-AP15 enhances TRAIL-induced cell death in HNSCC cells. **A** CCK8 cell viability analysis of HNSCC cell lines treated with increasing doses of b-AP15 and/or TRAIL for 48 h. Values below the combination are Combination Indices (CI) as described in the text. **B** Colony formation assay of HNSCC cell lines treated with b-AP15 (250 nM) and/or TRAIL (50 ng/mL) for 24 h. Representative images are shown with quantification below. **C** Annexin V analysis of HNSCC cell lines after treated with b-AP15 (250 nM) and/or TRAIL (50 ng/mL) for 24 and 48 h. Bars represent the means ± standard deviation. All experiments are representative of at least three biological replicates. NS not significant; *p < 0.05; **p < 0.01; ***p < 0.001 (Student’s *t* test).

To assess if b-AP15 treatment sensitises HNSCC cells to TRAIL, we first treated HNSCC cells with increasing doses of TRAIL alone or in combination with b-AP15 (Fig. 1A). We used TRAIL and b-AP15 at dose ranges used in HNSCC cells in previous studies [16, 29, 30]. In each cell line tested, TRAIL treatment alone had minimal effects on cell viability at the doses used (gray line). In line with our previous study, b-AP15 monotherapy significantly reduced cell viability (red line); however, the addition of exogenous TRAIL to b-AP15 significantly reduced cell viability in each cell line when compared with b-AP15 alone (dark red line). To determine if the combination treatment was synergistic, we calculated the combination index (CI); a CI less than 1 indicates a synergistic interaction [27]. Each combination resulted in a CI value < 0.6, indicating synergism between b-AP15 and TRAIL treatment in each HNSCC cell line tested.

To further assess the combination of b-AP15 and TRAIL treatment, we performed colony formation assays. As previously shown, b-AP15 treatment significantly reduced colony formation in each cell line tested (Fig. 1B). Additionally, TRAIL treatment alone resulted in a small, but significant reduction in colony formation ability in HPV- UMSCC1 and 22A cells, but not in HPV + UMSCC47 cells. Importantly, treatment of b-AP15 and TRAIL lead to a further decrease in colony formation when compared to b-AP15 alone (Fig. 1B). Furthermore, combination treatment induced higher levels of apoptosis, at both 24 and 48 h, when compared to b-AP15 alone (Fig. 1C). Together, these data suggest that b-AP15 treatment sensitises HNSCC cells to TRAIL treatment.

### Enhanced DR5 expression is required for b-AP15-mediated TRAIL sensitivity

**Fig. 2:**
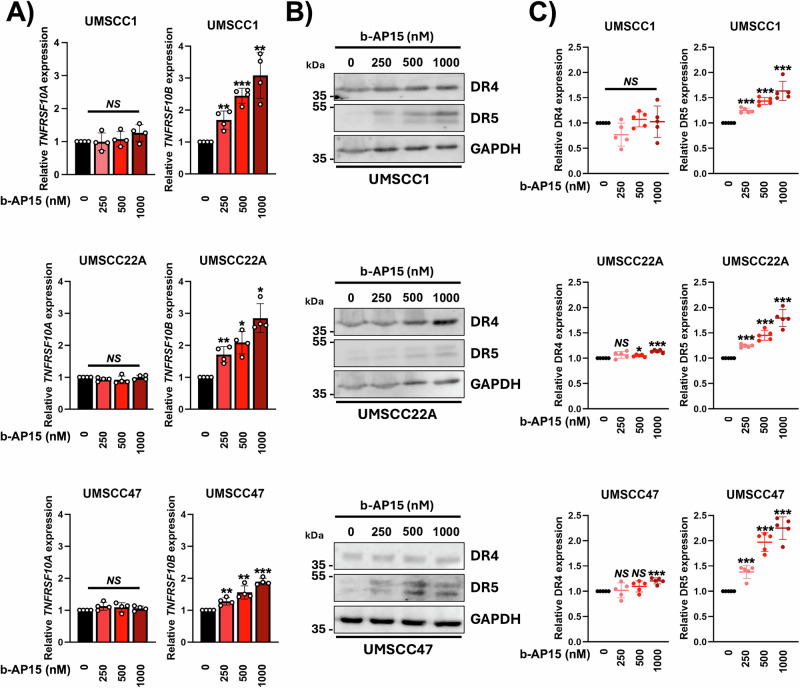
b-AP15 enhances DR5/TRAILR2 expression. **A** RT-qPCR analysis of *TNFRSF10A* and *TNFRSF10B* expression in HNSCC cell lines treated with increasing doses of b-AP15 for 24 h. *GAPDH* was used as a loading control. **B** Representative western blot of the expression of DR4/TRAILR1 and DR5/TRAILR2 in HNSCC cell lines treated with increasing doses of b-AP15 for 24 h. GAPDH was used as a loading control. **C** Flow cytometry analysis of DR4/TRAILR1 and DR5/TRAILR2 expression in HNSCC cell lines treated with increasing doses of b-AP15 for 24 h. All experiments are representative of at least three biological replicates. NS not significant; *p < 0.05; **p < 0.01; ***p < 0.001 (Student’s *t* test).

**Fig. 3:**
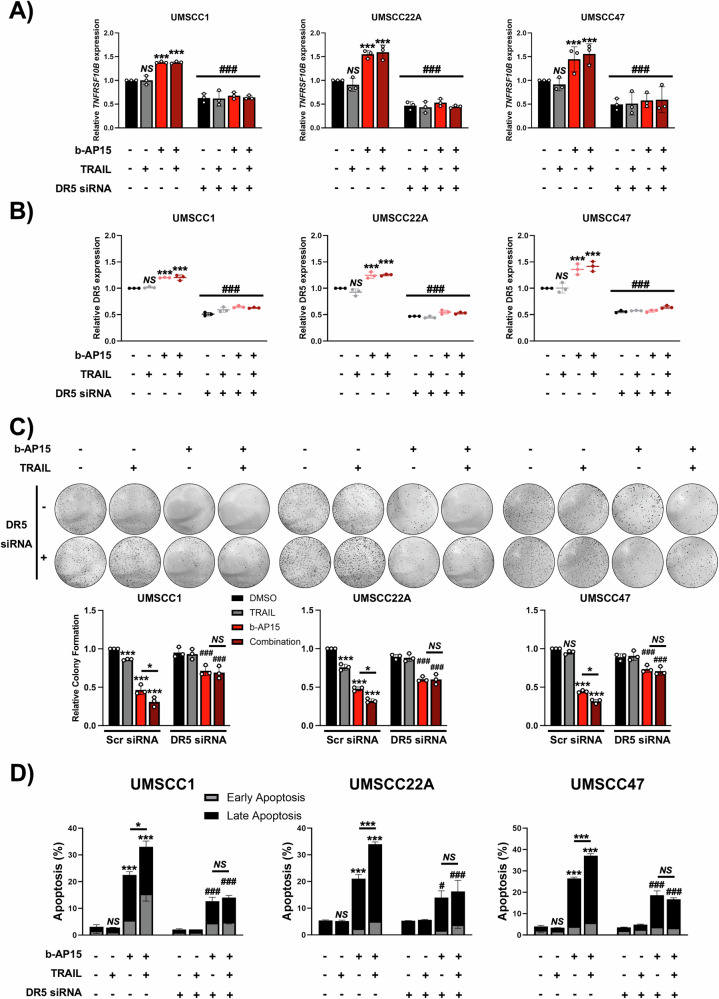
Enhanced DR5 expression is required for b-AP15-mediated TRAIL sensitivity. **A** RT-qPCR analysis of *TNFRSF10B* expression in HNSCC cell lines after transfection with a pool of four *TNFRSF10B* specific siRNAs for 72 h. Cells were treated with b-AP15 and/or TRAIL 48 h before analysis. *GAPDH* was used as a loading control. ^#^indicates that the statistical analysis is compared to the DMSO control in the Scr control group. **B** Flow cytometry analysis of DR5/TRAILR2 expression in HNSCC cell lines after transfection with a pool of four *TNFRSF10B* specific siRNAs for 72 h. Cells were treated with b-AP15 and/or TRAIL 24 h before analysis. ^#^indicates that the statistical analysis is compared to the DMSO control in the Scr control group. **C** Colony formation assay of HNSCC cell lines after transfection with a pool of four *TNFRSF10B* specific siRNAs for 72 h. Cells were treated with b-AP15 and/or TRAIL 24 h before analysis. Representative images are shown with quantification below. ^#^indicates that the statistical analysis is compared to the corresponding condition in the Scr control group. **D** Annexin V analysis of HNSCC cell lines after transfection with a pool of four *TNFRSF10B* specific siRNAs for 72 h. Cells were treated with b-AP15 and/or TRAIL 48 h before analysis. ^#^indicates that the statistical analysis is compared to thecorresponding condition in the Scr control group. All experiments are representative of at least three biological replicates. NS not significant; */^#^*p* < 0.05; **/^##^*p* < 0.01^;^ ***/^###^*p* < 0.001 (Student’s *t* test).

To determine the mechanism for how b-AP15 treatment enhances TRAIL sensitivity, we first looked at the expression of the two main TRAIL receptors, DR4/TRAILR1 and DR5/TRAILR2 [17]. Increasing doses of b-AP15 did not affect the mRNA expression of *TNFSRF10A*, the gene encoding for DR4 (Fig. 2A, left); however, b-AP15 increased the expression of *TNFSRF10B*, the gene encoding for DR5, in a dose-dependent manner in each HNSCC cell line tested (Fig. 2A, right). Similarly, increasing doses of b-AP15 induced DR5 protein expression, both total protein (Fig. 2B) and expression at the cell surface (Fig. 2C). DR4 protein expression was increased at higher doses of b-AP15 in UMSCC22A and 47 cells, but this was not to the same levels as DR5. Together, these data suggest that b-AP15 induces the expression of TRAIL receptors, predominantly DR5, at the transcriptional level, resulting in enhanced cell surface expression in HNSCC cells. To investigate if enhanced DR5 expression is responsible for b-AP15-mediated TRAIL sensitisation, we depleted DR5 expression in HNSCC cells using siRNA. DR5 expression was significantly reduced at both the mRNA and cell surface level (Fig. 3A, B). Depletion of DR5 abrogated the proliferation defects and reduced the level of apoptosis observed upon combination treatment of b-AP15 and TRAIL (Fig. 3 C, D). Interestingly, DR5 depletion also reduced the proliferation defects and apoptosis levels upon b-AP15 monotherapy. In validation of this, treatment with b-AP15 also sensitised HNSCC cells to treatment with a DR5 agonist antibody in a synergistic manner (CI < 0.65 in each HNSCC cell line tested), confirming the role of DR5 in b-AP15-mediated TRAIL sensitisation (Supplementary Fig. 1A–C). Additionally, DR5-mediated apoptosis may also play a role in the apoptotic functions of b-AP15 monotherapy.

### b-AP15-mediated DR5 expression and TRAIL sensitisation requires ROS induction

**Fig. 4:**
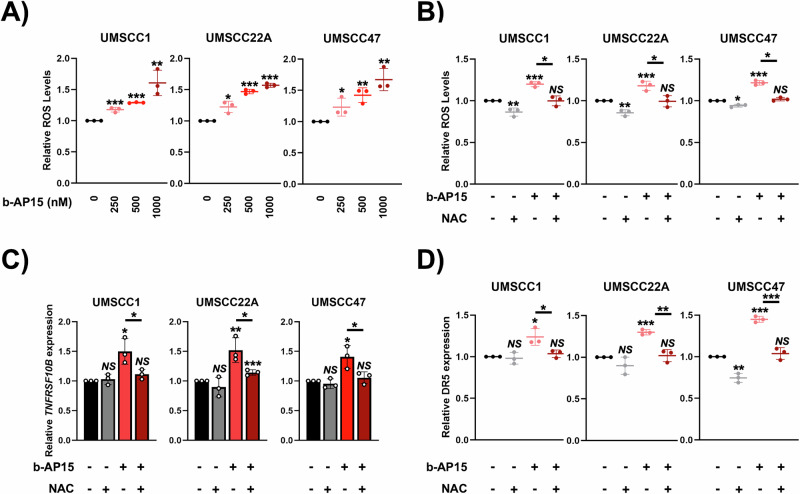
b-AP15-mediated DR5 expression requires ROS induction. **A** Flow cytometry analysis ROS in HNSCC cell lines treated with increasing doses of b-AP15 for 24 h. The fluorescent probe DCFH-DA was added to cells for the detection of ROS. **B** Flow cytometry analysis of ROS in HNSCC cell lines treated with b-AP15 for 24 h. NAC or DMSO control was added 2 h before b-AP15 treatment. **C** RT-qPCR analysis of *TNFRSF10B* expression in HNSCC cell lines treated with b-AP15 for 24 h. NAC or DMSO control was added 2 h before b-AP15 treatment. *GAPDH* was used as a loading control. **D** Flow cytometry analysis of DR5/TRAILR2 expression in HNSCC cell lines treated with b-AP15 for 24 h. NAC or DMSO control was added 2 h before b-AP15 treatment. All experiments are representative of at least three biological replicates. NS not significant; *p < 0.05; **p < 0.01; ***p < 0.001 (Student’s *t* test).

Previous studies have suggested that the induction of reactive oxygen species (ROS) can induce the expression of DR5 [31, 32]. Given that we previously demonstrated that b-AP15 inhibits NFκB activity [16] and that NFκB activity can prevent ROS induction [33, 34], we investigated if ROS are involved in b-AP15-mediated DR5 expression in HNSCC cells. Treatment with b-AP15 resulted in a dose-dependent increase in ROS production in HNSCC cells, as determined by fluorescence microscopy and quantified by flow cytometry (Fig. 4A, B). To determine if b-AP15-mediated ROS are required for the induction of DR5, we pre-treated cells with N-Acetyl-L-cysteine (NAC), a potent scavenger of ROS. As expected, pre-treatment of cells with NAC abrogated b-AP15-mediated ROS production (Fig. 4C, D). Additionally, NAC pre-treatment prevented b-AP15-mediated DR5 expression, at both the mRNA level and at the cell surface (Fig. 4E, F). Given that NAC pre-treatment prevented b-AP15-mediated DR5 upregulation, we investigate if ROS were required for b-AP15-induced apoptosis and TRAIL sensitisation. Pre-treatment with NAC significantly reduced the proliferation defects observed after b-AP15 treatment, as well as reducing the level of apoptosis (Supplementary Fig. 2A and B). Furthermore, NAC pre-treatment prevented the ability of b-AP15 to sensitise HNSCC cells to TRAIL treatment Supplementary Fig. 2C and D. Together, these data demonstrate that b-AP15-mediated ROS induction is required for DR5 upregulation and TRAIL sensitisation.

### JNK signalling contributes to DR5 expression and TRAIL sensitization

**Fig. 5:**
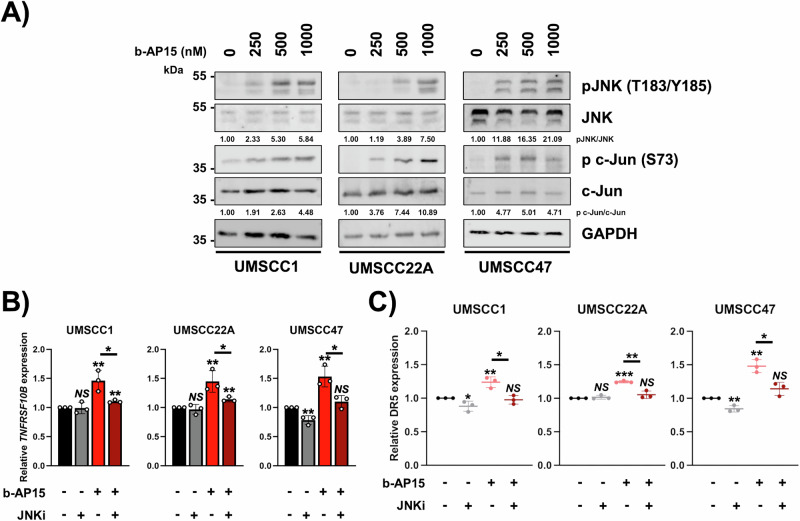
JNK activation is required for b-AP15-mediated DR5 expression. **A** Representative western blot of phosphorylated and total JNK, and phosphorylated and total c-Jun in HNSCC cell lines treated with increasing doses of b-AP15 for 24 h. GAPDH was used as a loading control. **B** RT-qPCR analysis of *TNFRSF10B* expression in HNSCC cell lines treated with b-AP15 for 24 h. JNK inhibitor 8 (JNK-IN-8) or DMSO control was added 3 h before b-AP15 treatment. *GAPDH* was used as a loading control. **C** Flow cytometry analysis of DR5/TRAILR2 expression in HNSCC cell lines treated with b-AP15 for 24 h. JNK-IN-8 or DMSO control was added 3 h before b-AP15 treatment. *GAPDH* was used as a loading control. All experiments are representative of at least three biological replicates. NS not significant; *p < 0.05; **p < 0.01; ***p < 0.001 (Student’s *t* test).

The production of ROS can induce multiple pro-apoptotic pathways, including the JNK signalling pathway [35]. Moreover, NFκB activity can inhibit the TNF-induced activation of JNK and JNK activity can induce the expression of DR5 [31, 36, 37]. Therefore, we hypothesised that b-AP15 treatment may induce JNK activity and subsequent DR5 expression. Treatment of HNSCC cells with b-AP15 activated the JNK pathway in a dose-dependent manner, as evidenced by increased JNK phosphorylation and c-Jun phosphorylation, a well characterised substrate of JNK [38, 39] (Fig. 5A). To determine if JNK activity is required for b-AP15-mediated TRAIL sensitivity, we pre-treated HNSCC cells with JNK-IN-8, a specific, covalent JNK inhibitor [40]. Pre-treatment with JNK-IN-8 significantly reduced b-AP15-mediated induction of DR5 expression, both at the transcriptional level and at the cell surface, suggesting that JNK activity contributes to b-AP15-mediated DR5 expression (Fig. 5B, C). To confirm if JNK activity contributed to b-AP15-mediated TRAIL sensitisation, cells were pre-treated with JNK-IN-8 before b-AP15 treatment. JNK inhibition significantly reduced b-AP15 induced proliferation defects and apoptosis induction, suggesting that JNK induction plays a role in the anti-tumour effects of b-AP15 in HNSCC cells (Supplementary Fig. 3A and B). Additionally, JNK inhibition abolished b-AP15-mediated TRAIL sensitisation (Supplementary Fig. 3C and D). These data demonstrate that JNK activity contributes to b-AP15-mediated DR5 upregulation and enhanced TRAIL sensitisation.

### XIAP downregulation contributes to b-AP15-mediated DR5 induction

**Fig. 6:**
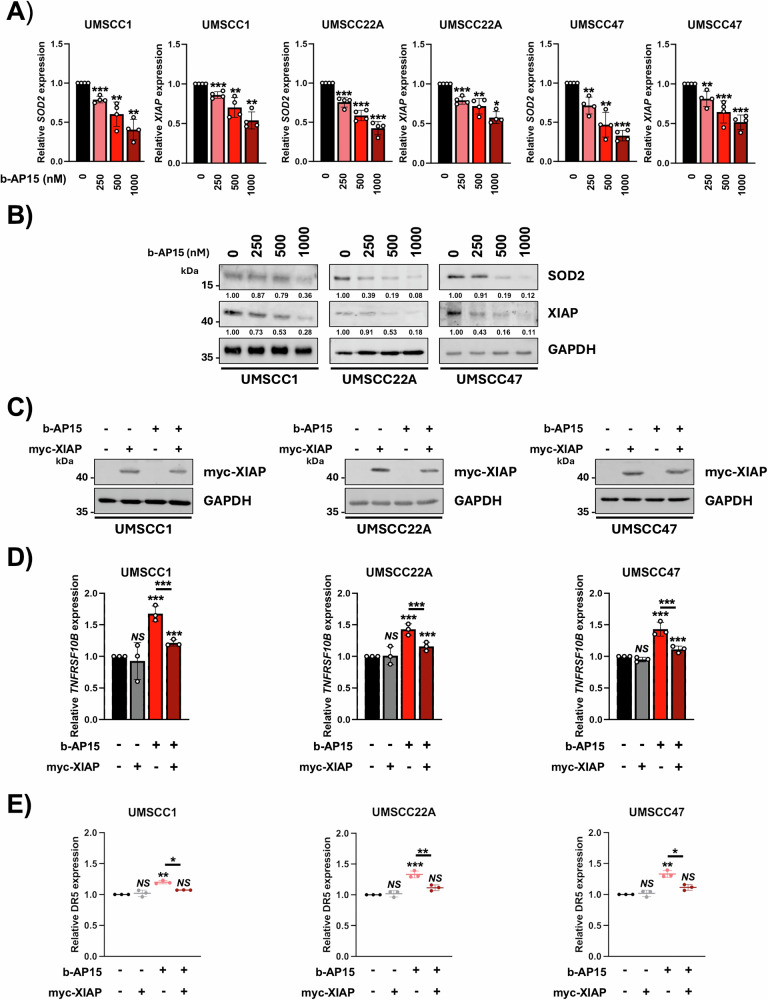
Downregulation of XIAP contributes to b-AP15-mediated DR5 induction. **A** RT-qPCR analysis of *SOD2* and *XIAP* expression in HNSCC cell lines treated with increasing doses of b-AP15 for 24 h. *GAPDH* was used as a loading control **B** Representative western blot of SOD2 and XIAP expression in HNSCC cell lines treated with increasing doses of b-AP15 for 24 h. GAPDH was used as a loading control. **C** Representative western blot of XIAP expression in HNSCC cell lines transfected with myc-XIAP for 48 h. Lysates were probed for myc-XIAP expression. GAPDH was used as a loading control. **D** RT-qPCR analysis of *TNFRSF10B* expression in HNSCC cell lines transfected with myc-XIAP. After 24 h, cells were treated with b-AP15 and analysed after another 24 h. *GAPDH* was used as a loading control. **E** Flow cytometry analysis of DR5/TRAILR2 expression in HNSCC cell lines transfected with myc-XIAP. After 24 h, cells were treated with b-AP15 and analysed after another 24 h. All experiments are representative of at least three biological replicates. NS not significant; *p < 0.05; **p < 0.01; ***p < 0.001 (Student’s *t* test).

**Fig. 7:**
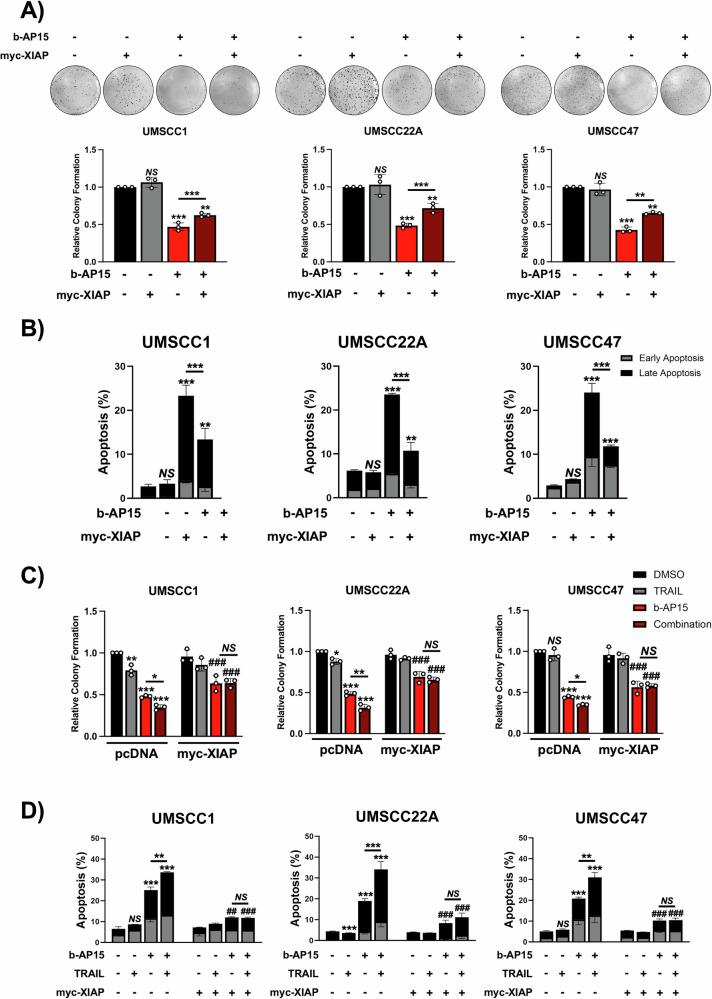
XIAP regulates b-AP15 mediated apoptosis and TRAIL sensitivity. **A** Colony formation assay of HNSCC cell lines after transfection with myc-XIAP for 48 h. Cells were treated with b-AP15 24 h before harvest. Representative images are shown with quantification below. **B** Annexin V analysis of HNSCC cell lines after transfection with myc-XIAP for 48 h. Cells were treated with b-AP15 24 h before harvest. **C** Colony formation assay of HNSCC cell lines after transfection with myc-XIAP for 48 h. Cells were treated with b-AP15 and/or TRAIL 24 h before harvest. Representative images are shown with quantification below. ^#^indicates that the statistical analysis is compared to the corresponding condition in the pcDNA control group. **D** Annexin V analysis of HNSCC cell lines after transfection with myc-XIAP for 48 h. Cells were also treated with b-AP15 and/or TRAIL. ^#^indicates that the statistical analysis is compared to the corresponding condition in the pcDNA control group. All experiments are representative of at least three biological replicates. NS not significant; *p < 0.05; **p < 0.01; ***p < 0.001 (Student’s *t* test).

Our recent study demonstrated that b-AP15 reduced NFκB activity and NFκB-dependent gene expression [16]. Several NFκB-dependent genes have been shown to supress ROS induction and inhibit JNK activity, including *SOD2* [41] and *XIAP* [42]. To investigate if the reduced expression of NFκB-dependent genes contributes to b-AP15-mediated TRAIL sensitisation, we first examined the expression of *SOD2* and *XIAP* by RT-qPCR in HNSCC cells treated with b-AP15. In each cell line tested, b-AP15 reduced *SOD2* and *XIAP* expression in a dose-dependent manner (Fig. 6A). Furthermore, this also resulted in a dose-dependent decrease in SOD2 and XIAP protein levels in these cells (Fig. 6B). XIAP is a member of the inhibitor of apoptosis family of proteins (IAP), which have been shown to be promising clinical targets in HNSCC [43–47]. We therefore investigated the role of XIAP in the cytotoxic effects of b-AP15. XIAP over-expression significantly reduced b-AP15-mediated DR5 induction, both transcriptionally and at the cell surface (Fig. 6C–E), demonstrating that b-AP15-mediated downregulation of XIAP expression plays a role in the increased DR5 expression observed upon b-AP15 treatment. We next assessed if XIAP contributes to b-AP15 mediated apoptosis and TRAIL sensitisation in HNSCC cells. XIAP over-expression significantly reduced the anti-proliferative and pro-apoptotic effects of b-AP15 (Fig. 7A, B). Moreover, XIAP over-expression abolished b-AP15-induced TRAIL sensitivity, demonstrating that XIAP plays a key role in mediating TRAIL/DR5 signalling in HNSCC cells (Fig. 7C, D).

### Combination treatment of b-AP15 with IAP antagonists is synergistic in HNSCC in vitro

**Fig. 8:**
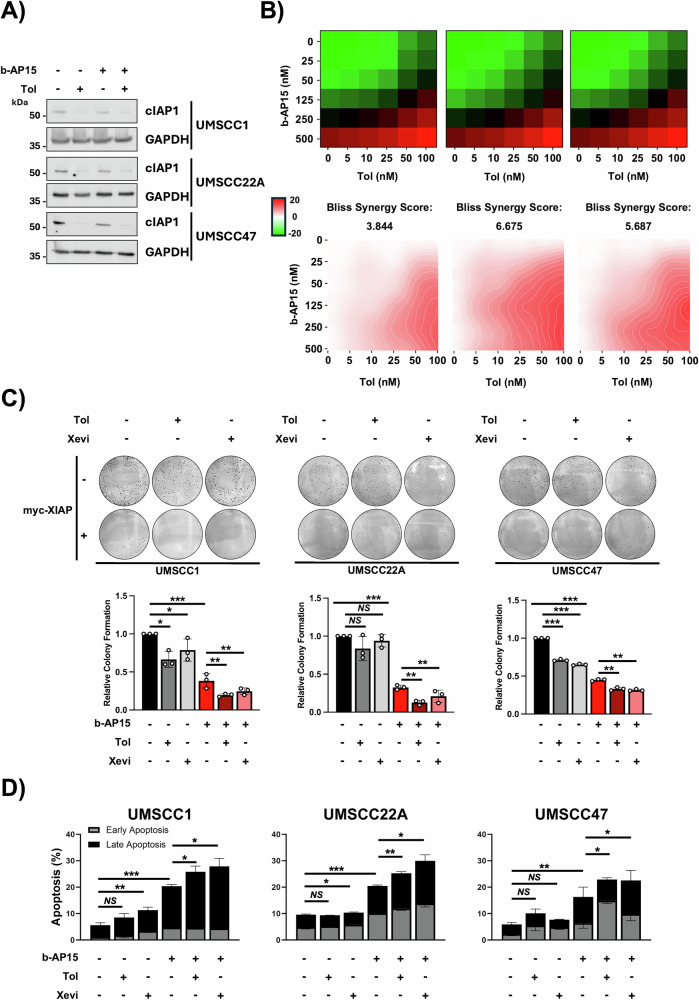
Combination treatment of b-AP15 with IAP mimetics is synergistic in HNSCC in vitro. **A** Representative western blot of cIAP1 expression in HNSCC cell lines treated with b-AP15 and/or Tolinapant (Tol) for 48 h. GAPDH was used as a loading control. **B** Synergism analysis of HNSCC cell lines treated with b-AP15 and/or Tol for 48 h. Cell viability heatmaps are shown at the top; Bliss synergy maps are shown below. **C** Colony formation assay of HNSCC cell lines treated with b-AP15 and/or Tol or Xevinapant (Xevi) for 48 h. Representative images are shown with quantification below. **D** Annexin V analysis of HNSCC cell lines treated with b-AP15 and/or Tol or Xevi for 48 h. All experiments are representative of at least three biological replicates. NS not significant; *p < 0.05; **p < 0.01; ***p < 0.001 (Student’s *t* test).

Given that our data suggests that XIAP plays a key role in regulating b-AP15-mediated anti-proliferative and pro-apoptotic effects, we hypothesised that combinatory treatment with b-AP15 and an XIAP inhibitor may be synergistic in HNSCC cells. To test this, we used two IAP antagonists that have been shown to inhibit the function of XIAP and are currently in clinical trials for HNSCC, Tolinapant/ASTX660 and Xevinapan/Debio 1143 [30, 44, 48, 49]. We first assessed cIAP1 levels after treatment by western blot, a marker for on target efficacy as IAP antagonists induce cIAP1 degradation [50]. As expected, both Tolinapant (Tol) and Xevinapant (Xevi) reduced cIAP1 levels by over 90%, whereas b-AP15 treatment resulted in a 40–50% reduction in cIAP1 levels (Fig. 8A and Supplementary Fig. 4A). XIAP levels remained unchanged by IAP antagonist treatment, consistent with previous data demonstrating that these small molecules disrupt the interaction between XIAP and caspases 3, 7 and 9 [50]. To assess potential drug synergy between b-AP15 and IAP antagonists, we performed dose matrix experiments and assessed potential drug synergy using the Bliss independence model as in our previous study [51]. An overall negative Bliss synergy score indicates antagonism, whereas a positive Bliss synergy score indicates synergy. Heatmaps indicated that combination treatment between b-AP15 and Tolinapant was mildly synergistic in each HNSCC cell line tested, particularly at the higher doses of Tolinapant, with Bliss Synergy scores between 3.8 and 6.7 (Fig. 8B). Combination treatment between b-AP15 and Xevinapant demonstrated greater synergy at lower IAP antagonist doses, with higher overall Bliss Synergy scores between 4.5 and 7.9 (Supplementary Fig. 4B). Combination treatments also resulted in a greater reduction in colony formation when compared to either inhibitor alone (Fig. 8C) and a small but significant increase in apoptosis (Fig. 8D). Altogether, these data suggest that combination treatment between b-AP15 and IAP antagonists is moderately synergistic in HNSCC cells, demonstrating greater anti-proliferative effects than either b-AP15 or IAP antagonist alone.

## Discussion

As an essential regulatory mechanism that controls several critical cellular processes, protein ubiquitination is a promising target in oncology, with deubiquitinases of particular interest due to their well-defined active site [52–54]. Our previous study demonstrated that inhibition of the proteasome-associated deubiquitinases USP14 and UCHL5 using the small molecule b-AP15 significantly reduced the proliferation and survival of HNSCC cells, both in vitro and in vivo [16]. USP14 promoted the activation of the canonical NFκB pathway, a critical driver of proliferation, survival, and therapeutic resistance in HNSCC [7, 8]. The inhibition of NFκB activity by b-AP15 sensitised HNSCC cells to TNF-induced cell death, a key mediator of radiation mediated cytotoxicity. Given that TRAIL is cancer cell-selective when compared with TNF [55], whether b-AP15 could enhance the cytotoxic effects of TRAIL was of interest.

TRAIL resistance has been related to differing genomic alterations in the cell death pathways in cancers, including HNSCC [56–59]. Findings from TCGA and other studies have revealed that around 40% of HNSCC harbour genomic alterations in cell death pathways [13, 14]. FADD and IAP1/2 (products of the *BIRC2/BIRC3* genes) are frequently co-amplified in HPV- HNSCC, promoting tumour growth and survival [13, 45]. Furthermore, 10% of HNSCC patients form a mutually exclusive subset with mutations in Caspase 8 (*CASP8)* [59]. Multiple studies have shown that alterations in these pathways can render cancer cells resistance to TRAIL through various mechanisms, such as the activation of NFκB signalling and the destabilisation of Caspase 8. In particular, TRAIL-inducible NFκB activity has limited the efficacy of TRAIL monotherapies in cancer [60]. Thus, inhibition of NFκB activity offers the potential as a TRAIL sensitising agent. Previous studies have shown that HNSCC cells line have variable responses to TRAIL treatment alone, with HPV + HNSCC cell lines showing resistance to TRAIL treatment when compared with HPV- HNSCC cell lines [57]. Furthermore, proteasome inhibition by MG132 or Bortezomib treatment sensitised both HPV+ and HPV- HNSCC cells to TRAIL treatment [57, 61]. Our data demonstrates that b-AP15 also sensitises HNSCC cell lines to TRAIL, in addition to its ability to sensitise HNSCC to TNFα and radiation treatment and consistent with data in other cell lines [16, 62, 63]. Of note, the TRAIL doses used here are 100-1000x higher than physiological concentrations to overcome resistance that is often observed in cancer cell lines. Thus, in vivo studies are essential to determine if the effects observed here are also observed at clinically relevant doses in more physiologically relevant models. Our data also further expand understanding on the mechanisms of b-AP15 action. We identified that b-AP15 upregulates the expression of the TRAIL receptor DR5, consistent with a previous study [63]. Interestingly, this appears to be specific to DR5 over the other TRAIL receptor DR4 and DR5 is required for the ability of b-AP15 to sensitise HNSCC cell lines to TRAIL treatment (Fig. 3). Further studies will be required to identify if b-AP15 effects other components of this signalling pathway, such as FADD, cFLIP or Caspase 8, in HNSCC cells, although these components were unaffected in a previous study [63]. Several studies have identified regulatory pathways controlling the expression of DR5 [64]. The induction of ROS has been implicated in regulating DR5 expression in multiple cancers [31, 32]; furthermore, NFκB is known to control the expression of antioxidant genes that prevent the TNF-induced accumulation of ROS [41]. Given our previous data that b-AP15 inhibits NFκB activity in HNSCC, our data here shows that b-AP15 decreases the expression of SOD2, a critical NFκB-dependent antioxidant gene. b-AP15 also resulted in a dose-dependent accumulation of ROS, consistent with its effects in other cancer cell lines [65–67]. While ROS induction appears to be essential for b-AP15-mediated DR5 expression and the sensitisation to TRAIL, further studies are required to confirm if the downregulation of SOD2 is the causal factor in the accumulation of ROS induced by b-AP15 in HNSCC cells. ROS are well known to play a role in the activation of JNK, a pro-apoptotic pathway that is also negatively regulated by NFκB activity [31, 33, 35, 36]. In line with this, b-AP15 led to a dose-dependent activation of JNK and inhibition of JNK activity reduced DR5 expression, the anti-proliferative and pro-apoptotic effects of b-AP15 and the sensitisation to TRAIL. Inhibition of JNK had no effect on ROS levels (data not shown), suggesting that JNK activation is a downstream effect of ROS accumulation, in line with other studies [68]. This suggests that ROS-mediated JNK activity is required for b-AP15-mediated DR5 upregulation, consistent with other studies showing that DR5 can be regulated via ROS/JNK signalling [31, 32, 37]. Whether or not c-Jun plays a direct role in the regulation of DR5 requires further assessment.

Recent studies have demonstrated that IAP antagonists (also called SMAC mimetics) can sensitise HNSCC cell lines to TRAIL treatment [30, 45, 69]. As IAP antagonists induce the degradation of IAP proteins such as cIAP1/2 and can inhibit the activity of XIAP [50], we hypothesised that combination treatment of b-AP15 and IAP antagonists may be synergistic in HNSCC cells. We focused on XIAP, as it is highly upregulated in several cancers and is a primary mediator of NFκB-mediated JNK inhibition in response to TNFα [20, 36, 70, 71]. Furthermore, XIAP depletion has been shown to sensitise cancer cells to TRAIL and has been suggested as a key regulator of TRAIL-resistance [72, 73]. XIAP was downregulated in a dose-dependent manner in HNSCC cells and restoration of XIAP expression significantly reduced DR5 expression and TRAIL sensitisation, suggesting that XIAP downregulation plays a key role in the cytotoxic effects of b-AP15 in HNSCC. Given this, combination treatment between b-AP15 and two well studied IAP antagonists that target XIAP, Tolinapant and Xevinapant, was mildly synergistic in HNSCC cells, demonstrating decreased colony formation potential and increased apoptosis (Fig. 8 and Supplementary Fig. 4). These data demonstrate the potential for combination treatments between b-AP15 and IAP antagonists, highlighting that targeting the ubiquitin system is a viable therapeutic option in HNSCC. This also expands our work on identifying possible combination treatments with IAP antagonists, as we have previously demonstrated with WEE1 inhibitors [74]. Further assessment of this combination of inhibitors is warranted, including other USP14 inhibitors and other XIAP-targeting IAP antagonists, as well as in vivo models to assess for toxicities and pre-clinical efficacy. Additionally, targeting XIAP via genetic means should be explored, as this has been shown to be a promising avenue [75, 76].

One interesting finding from this study, is that inhibition of ROS and/or JNK at least partially reduce the anti-proliferative and pro-apoptotic effects of b-AP15 alone. This suggests that the activation of ROS and JNK-dependent pathways are important mediators of the action of b-AP15, one of which is the upregulation of DR5 and the subsequent sensitisation to TRAIL. Furthermore, our data shows XIAP reduced the cytotoxic effects of b-AP15, suggesting that XIAP downregulation is an important function of b-AP15 in HNSCC cells. It would be of interest to see if inhibition of USP14 by other means (IU1-47 treatment or USP14 depletion by siRNA) results in the activation of a similar pathway (e.g. ROS induction, JNK activation and XIAP downregulation).

In summary, our data demonstrate that b-AP15 sensitises HNSCC cell lines to TRAIL treatment via the upregulation of DR5 expression in a manner dependent on ROS accumulation and JNK activation. Furthermore, we show that small molecules targeting XIAP are synergistic with b-AP15 in HNSCC cells in vitro. We believe this is the first demonstration of a synergistic combination between a E3 ligase inhibitor and a deubiquitinase inhibitor, cementing the promise of the ubiquitin system as a potential therapeutic target in oncology research. Pre-clinical development of small molecules targeting USP14 is needed, with combination treatments with other small molecules targeting cell death pathways such as IAP antagonists offering a promising avenue for future studies.

## Supplementary information

Supplemental Figures

## Data Availability

All data generated or analysed during this study are included in this published paper and its supplementary information files.
