# Supplementary material for: b-AP15 enhances TRAIL-induced cell death in HNSCC via the induction of ROS/JNK/DR5 signalling
Source: Cancer Gene Ther. 2026 May 2;33(5):533–46. doi: 10.1038/s41417-026-01038-3 (PMC13364667; doi:10.1038/s41417-026-01038-3)
Supplement: Supplementary file 1 — Supplemental Figures [file 41417_2026_1038_MOESM1_ESM.docx]

**
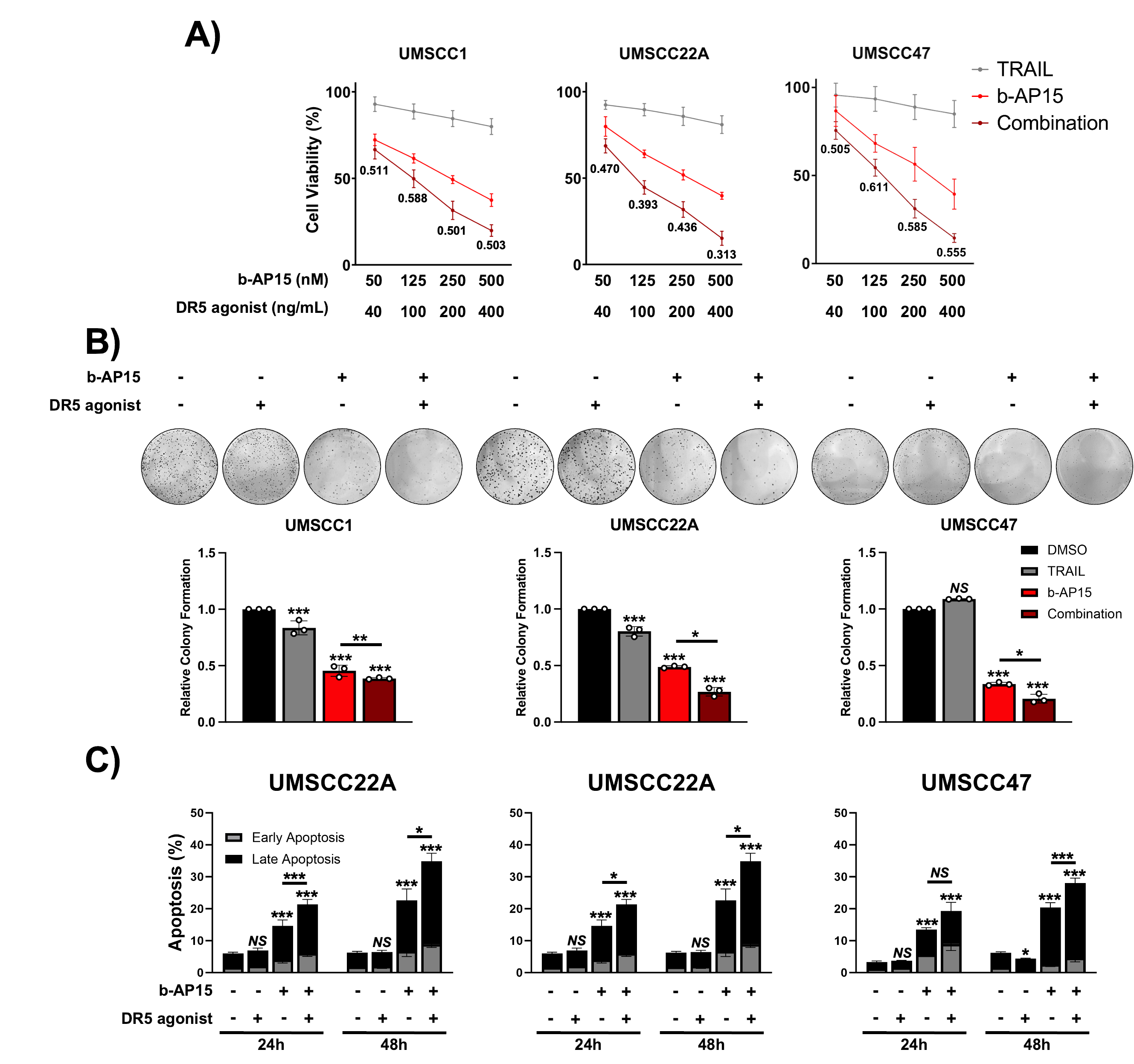
Supp Fig 1. b-AP15 enhances DR5/TRAILR2 agonist-induced cell death in HNSCC cells. A**CCK8 cell viability analysis of HNSCC cell lines after treatment with varying doses of b-AP15 and/or DR5/TRAILR2 agonist (hereafter DR5 agonist) for 48 h. Values below the combination are Combination Indices (CI) as described in the text.**B**Colony formation assay of HNSCC cell lines after treatment with DR5 agonist (200 ng/mL), b-AP15 (250 nM) or the combination for 48h. Representative images are shown with quantification below.**C** Annexin V analysis of HNSCC cell lines after treatment with DR5 agonist (200 ng/mL), b-AP15 (250 nM), or the combination for 24 and 48 h. Bars represent the means ± standard deviation. All experiments are representative of at least three biological replicates. NS not significant; **p*  <  0.05; ***p*  <  0.01; ****p*  <  0.001 (Student’s *t* test).

**
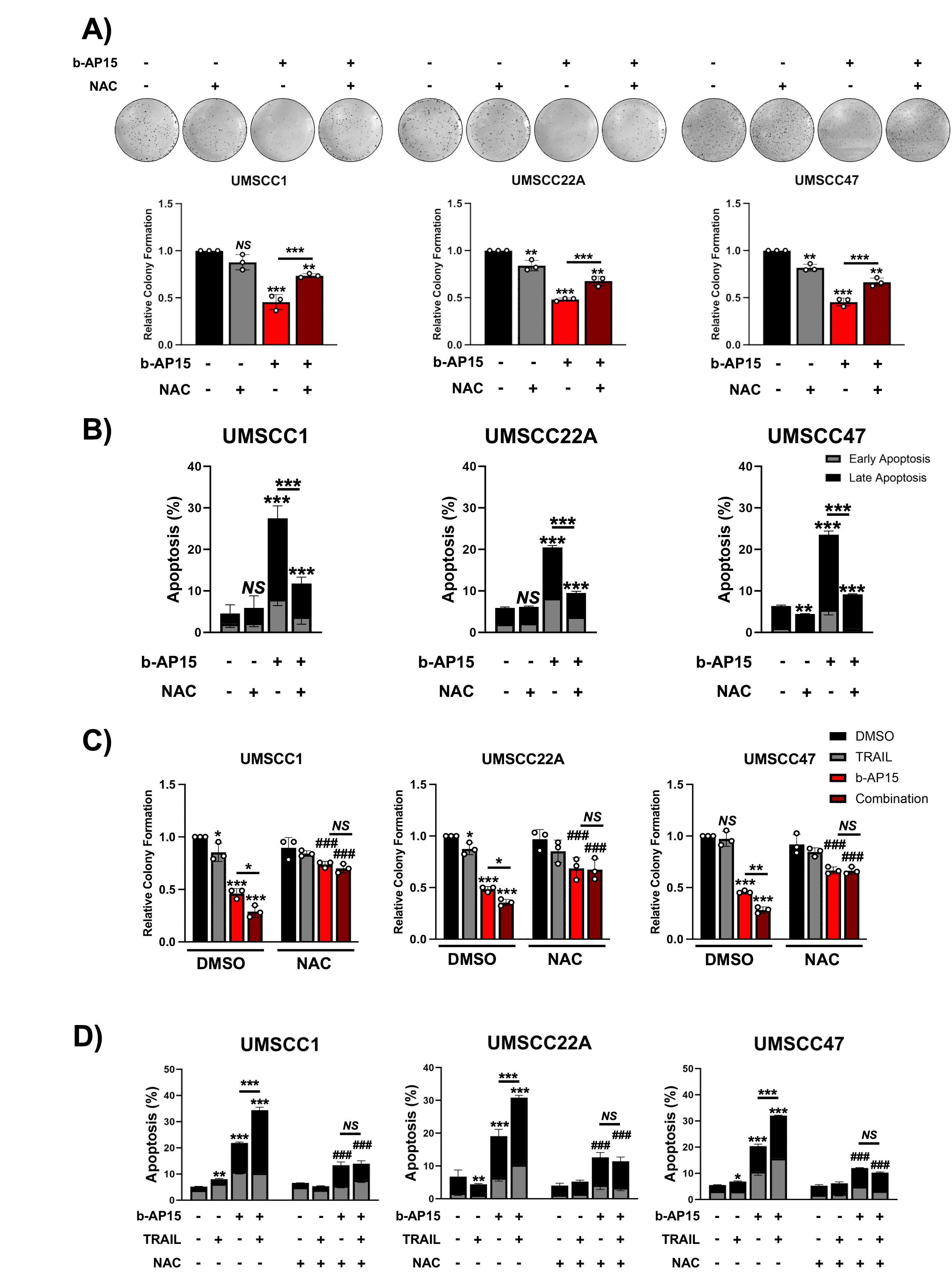
Supp Fig 2. ROS contribute to b-AP15-mediated apoptosis and TRAIL sensitivity b-AP15 enhances DR5/TRAILR2 agonist-induced cell death in HNSCC cells. A**Colony formation assay of HNSCC cell lines treated with b-AP15 for 24h. NAC or DMSO control was added 2 hours before b-AP15 treatment. Representative images are shown with quantification below. **B** Annexin V analysis of HNSCC cell lines treated with b-AP15 for 24h. NAC or DMSO control was added 2 hours before b-AP15 treatment. **C** Colony formation assay of HNSCC cell lines treated with b-AP15 and/or TRAIL for 24h. NAC or DMSO control was added 2 hours before b-AP15 treatment. Representative images are shown with quantification below. ^#^indicates that the statistical analysis is compared to the DMSO control in the DMSO control group. **D** Annexin V analysis of HNSCC cell lines treated with b-AP15 for 24h. NAC or DMSO control was added 2 hours before b-AP15 treatment. ^#^indicates that the statistical analysis is compared to the DMSO control in the DMSO control group. All experiments are representative of at least three biological replicates. NS not significant; **p*  <  0.05; ***p*  <  0.01; ****p*  <  0.001 (Student’s *t* test).

**
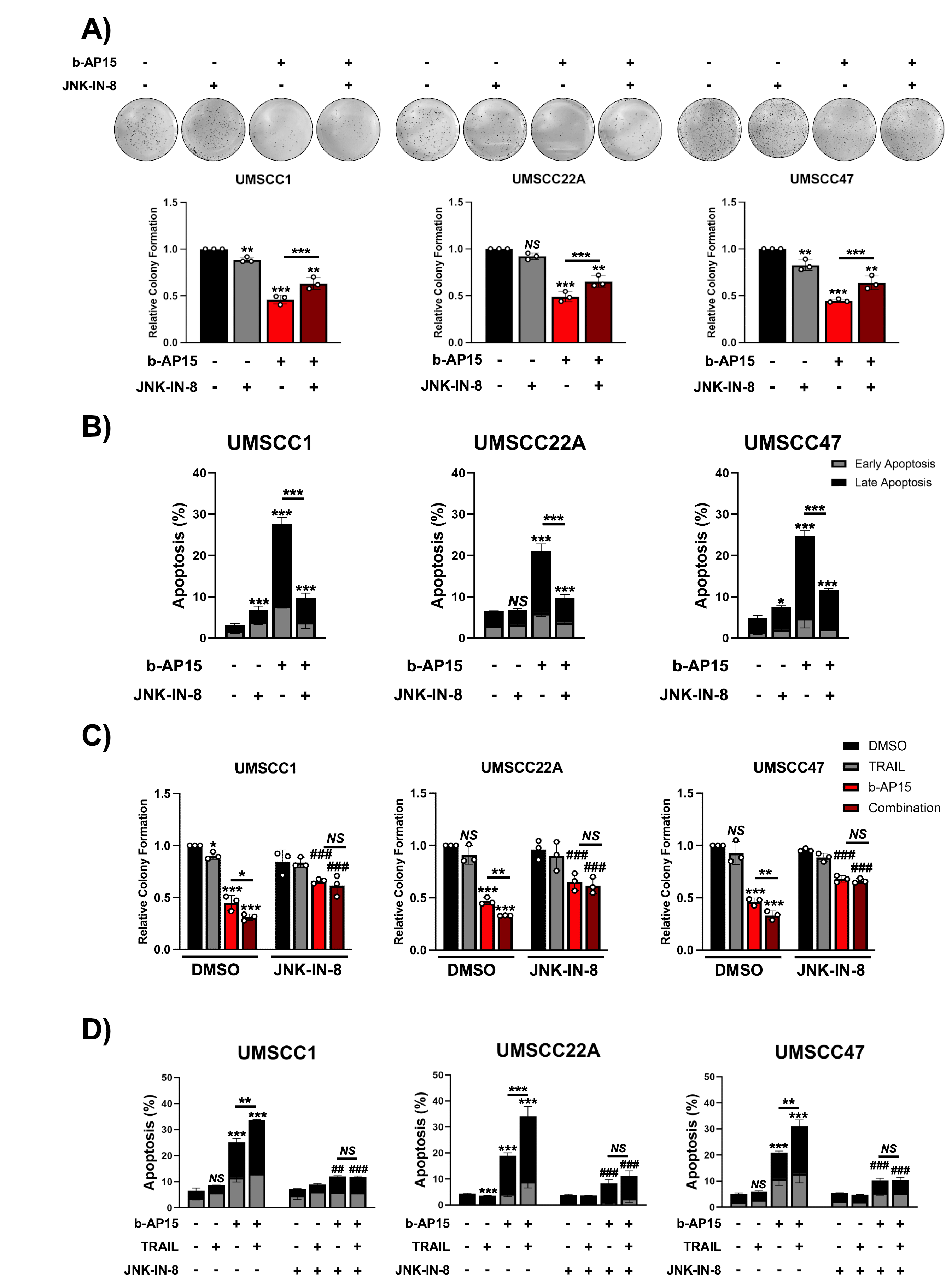
Supp Fig 3. JNK signaling contributes to b-AP15-mediated apoptosis and TRAIL sensitivity. A** Colony formation assay of HNSCC cell lines treated with b-AP15 for 24h. JNK-IN-8 or DMSO control was added 3 hours before b-AP15 treatment. Representative images are shown with quantification below. **B** Annexin V analysis of HNSCC cell lines treated with b-AP15 for 24h. JNK-IN-8 or DMSO control was added 3 hours before b-AP15 treatment. **C** Colony formation assay of HNSCC cell lines treated with b-AP15 and/or TRAIL for 24h. JNK-IN-8 or DMSO control was added 3 hours before b-AP15 treatment. Representative images are shown with quantification below. ^#^indicates that the statistical analysis is compared to the DMSO control in the DMSO control group. **D** Annexin V analysis of HNSCC cell lines treated with b-AP15 for 24h. JNK-IN-8 or DMSO control was added 3 hours before b-AP15 treatment. ^#^indicates that the statistical analysis is compared to the DMSO control in the DMSO control group. All experiments are representative of at least three biological replicates. NS not significant; **p*  <  0.05; ***p*  <  0.01; ****p*  <  0.001 (Student’s *t* test).

**
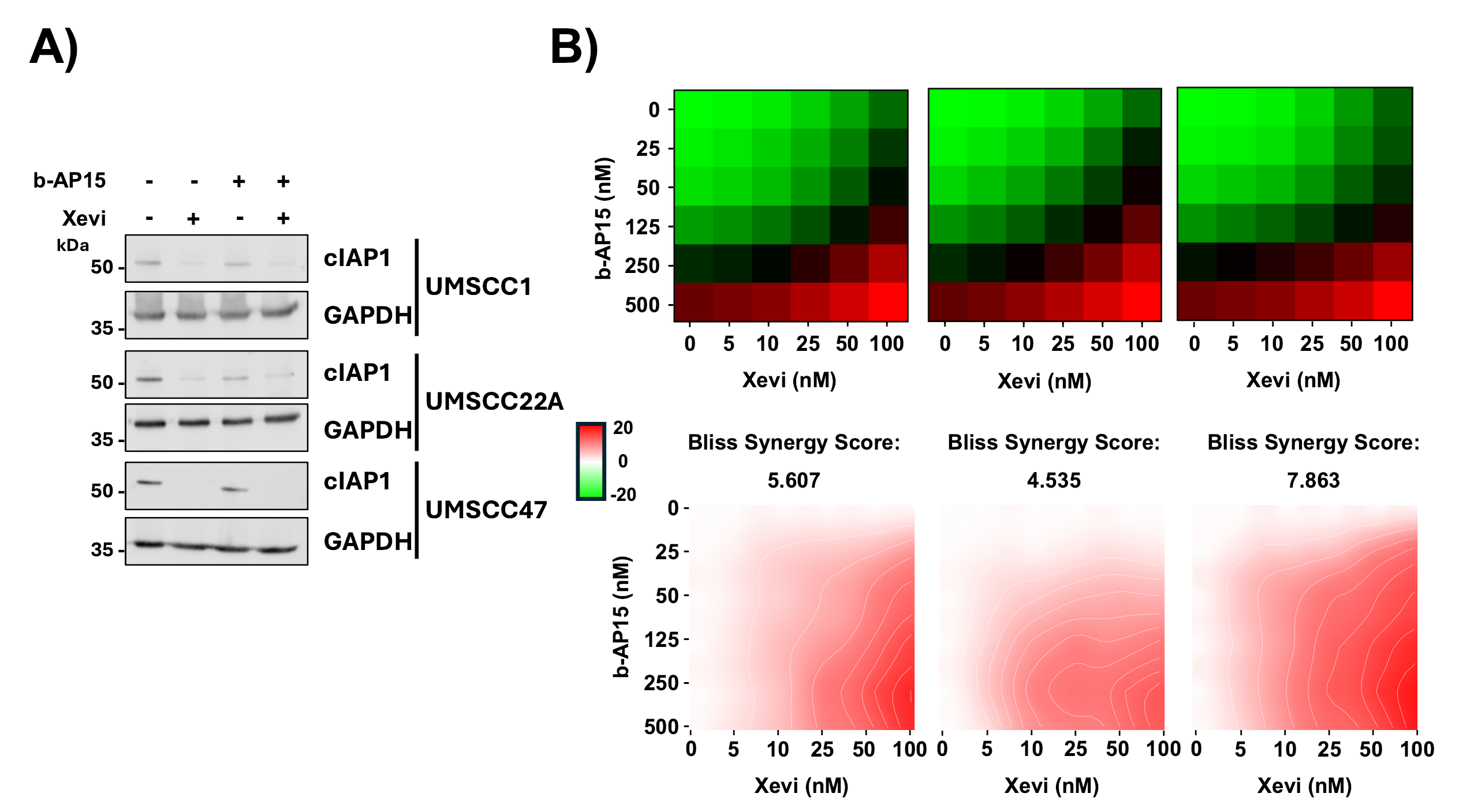
**

**Supp Fig 4. Combination treatment of b-AP15 with IAP mimetic Xevinapant is synergistic in HNSCC *in vitro*. A** Representative western blot of cIAP1 expression in HNSCC cell lines treated with b-AP15 and/or Xevinapant (Xevi) for 48h. GAPDH was used as a loading control. **B** Synergism analysis of HNSCC cell lines treated with b-AP15 and/or Xevi for 48h. Cell viability heatmaps are shown at the top; Bliss synergy maps are shown below.

**
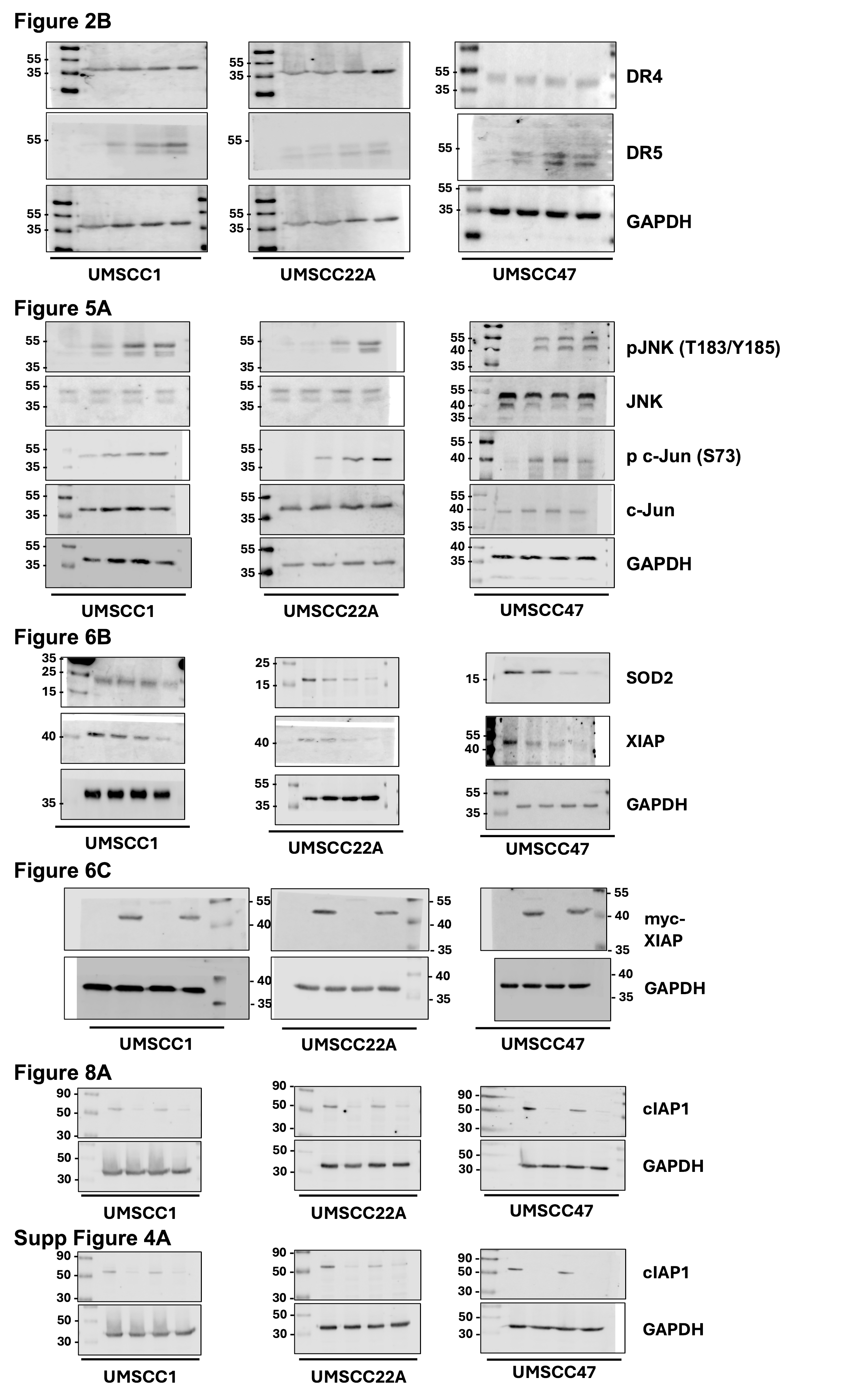
**

**Supplementary data.** Uncropped western blots.
